# Chromosomal Mapping of Repetitive DNAs in *Triportheus trifurcatus* (Characidae, Characiformes): Insights into the Differentiation of the Z and W Chromosomes

**DOI:** 10.1371/journal.pone.0090946

**Published:** 2014-03-14

**Authors:** Cassia Fernanda Yano, Juliana Poltronieri, Luiz Antonio Carlos Bertollo, Roberto Ferreira Artoni, Thomas Liehr, Marcelo de Bello Cioffi

**Affiliations:** 1 Departamento de Genética e Evolução, Universidade Federal de São Carlos, São Carlos, SP, Brazil; 2 Jena University Hospital, Friedrich Schiller University, Institute of Human Genetics, Jena, Thüringen, Germany; 3 Departamento de Biologia Estrutural, Molecular e Genética, Universidade Estadual de Ponta Grossa, Ponta Grossa, PR, Brazil; CNRS/University Lyon 1, France

## Abstract

Repetitive DNA sequences play an important role in the structural and functional organization of chromosomes, especially in sex chromosome differentiation. The genus *Triportheus* represents an interesting model for such studies because all of its species analyzed so far contain a ZZ/ZW sex chromosome system. A close relationship has been found between the differentiation of the W chromosome and heterochromatinization, with the involvement of different types of repetitive DNA in this process. This study investigated several aspects of this association in the W chromosome of *Triportheus trifurcatus* (2n = 52 chromosomes), including the cytogenetic mapping of repetitive DNAs such as telomeric sequences (TTAGGG)n, microsatellites and retrotransposons. A remarkable heterochromatic segment on the W chromosome was observed with a preferential accumulation of (CAC)_10_, (CAG)_10_, (CGG)_10_, (GAA)_10_ and (TA)_15_. The retrotransposons *Rex1* and *Rex3* showed a general distribution pattern in the chromosomes, and *Rex6* showed a different distribution on the W chromosome. The telomeric repeat (TTAGGG)n was highly evident in both telomeres of all chromosomes without the occurrence of ITS. Thus, the differentiation of the W chromosome of *T. trifurcatus* is clearly associated with the formation of heterochromatin and different types of repetitive DNA, suggesting that these elements had a prominent role in this evolutionary process.

## Introduction

Fishes show huge chromosome diversity with interspecific variation in diploid numbers, the presence or absence of sex and supernumerary chromosomes and other polymorphic conditions [1]. However, most fish species do not show differentiated sex chromosomes. In fact, approximately only 10% of studied species present heteromorphic sex chromosomes, with the female heterogamety representing the most common case [2]. Included in this group is the Neotropical genus *Triportheus*. This genus is widely distributed in South America and can be found from Colombia to Uruguay. Sixteen species have been described for this group [3]; thus far, all show a conserved karyotype with 2n = 52 and are composed of meta- submetacentric chromosomes together with a well-differentiated ZZ/ZW sex chromosome system [4]–[11].

High heterochromatin formation is clearly associated with W chromosome differentiation in *Triportheus,* similar to the majority of the ZW sex systems reported to date for fishes [reviewed in 12]. Among the different *Triportheus* species, the Z chromosome is conserved, but the W chromosome differs in size (from similar to the Z chromosome to much smaller) and in the amount and distribution of C-positive heterochromatin [7], [10]. Therefore, the evolutionary dynamics of sex chromosomes in *Triportheus* appear to be similar to those of higher vertebrates,considering that several DNA repeats accumulate in the sex-specific chromosome, which results in its heterochromatinization. In some groups, however, the sex-specific W chromosome can also be larger than the Z chromosome as a result of a huge heterochromatin amplification. Such amplification has been observed in some lizard [13] and fish species, remarkably including those from the *Leporinus* genus and the Parodontidae family reviewed in [12].

The close relationship between heterochromatin formation and sex chromosome differentiation is widely known. In fact, during sex chromosome evolution, selection favors the restriction of sex-determining loci to a chromosome of the sex pair through recombination suppression [14]. With the absence of recombination, repetitive sequences are amplified in the non-recombinant region, resulting in the heterochromatinization of the sex-specific chromosome, perhaps as a cellular defense against this amplification [15]. The heterochromatin of sex chromosomes has a complex composition that mainly contains *in tandem* repetitive sequences and interspersed elements, most of which are retrotransposons [16]. Thus, repetitive DNA sequences play an important role in the structural and functional organization of heterochromatin and are key elements for the understanding of sex chromosome differentiation in many vertebrate species [17], [18].

As in other vertebrates, the heterochromatin of the fish sex chromosomes is also enriched with repetitive DNA sequences, as shown by the isolation and mapping of several sex-specific repetitive DNAs in this group. Such data have proven to be important tools for clarifying the dynamic processes involved in sex chromosome differentiation and for understanding genome evolution in eukaryotes [reviewed in 19]. Therefore, in this study, we mapped 12 repetitive DNA sequences in the species *T. trifurcatus* to analyze the degree of repetitive DNA accumulation on the differentiated ZW chromosomes and their association with the evolution of this sex system. The results show that the differentiation of the W chromosome is clearly associated with the formation of heterochromatin and different types of repetitive DNA, suggesting that these elements had a prominent role in this evolutionary process.

## Materials and Methods

### Material Collection and Classical Analysis

Seventeen individuals of *Triportheus trifurcatus* (nine males and eight females) from the Araguaia River (Brazil) were analyzed. The specimens were caught using a hand-net, and after capture, the animals were placed in sealed plastic bags containing oxygen and clean water and transported to the research station. The experiments followed ethical protocols, and anesthesia with clove oil was administered prior to sacrificing the animals to minimize suffering. The process was approved by the FAPESP committee under no. 2012/01778-2, and the collecting permit was obtained from SISBIO under no. 10538-1. Mitotic chromosomes were obtained from the anterior portion of the kidney, according to Bertollo and colleagues [20]. In addition to the standard Giemsa staining, the C-banding method [21] was employed to detect C-positive heterochromatin.

### Probe Preparation

Oligonucleotide probes containing microsatellite sequences (CA)_15_, (CAA)_10_, (CAC)_10_, (CAG)_10_, (CAT)_10_, (CGG)_10_, (GA)_15_, (GAA)_10_ and (TA)_15_ were directly labeled with Cy5 during synthesis by Sigma (St. Louis, MO, USA), as described by Kubat and colleagues [22]. The retrotransposable elements Rex 1, 3 and 6 were obtained by polymerase chain reaction (PCR) [23]. The 18S rDNA probe, corresponding to a 1,400-bp segment of the 18S rRNA gene, was obtained via PCR from the nuclear DNA according to Cioffi and colleagues [24]. All probes were labeled with DIG-11-dUTP using DIG-Nick-translation Mix (Roche, Mannheim, Germany) and were used for the fluorescence *in situ* hybridization (FISH) experiments.

### Fluorescence in situ Hybridization and Signal Detection

FISH was conducted as follows: slides with fixed chromosomes were maintained at 37°C for 1 hour and were then incubated with RNAse A (10 mg/ml) for 1 hour at 37°C in a moist chamber. The slides were then washed with PBS for 5 minutes, and 0.005% pepsin was applied for 10 minutes at room temperature. The slides were then washed again with PBS, and the material was fixed with 1% formaldehyde at room temperature for 10 minutes. After further washing, the slides were dehydrated for 2 minutes each in sequential baths of 70%, 85% and 100% ethanol. The chromosomal DNA was denatured in 70% formamide/2xSSC for 3 minutes at 72°C. The slides were dehydrated again in a cold ethanol series (70%, 85% and 100%) for 5 minutes each. The hybridization mixture, containing 100 ng denatured probe, 10 mg/ml dextran sulfate, 2xSSC and 50% formamide in a final volume of 30 µl, was heated to 95°C for 10 minutes and then applied to the slides. Hybridization was performed for a period of 16–18 hours at 37°C in a moist chamber. After hybridization, the slides were washed for 5 minutes with 2xSSC and then rinsed quickly in PBS. Signal detection was performed using anti-digoxigenin-FITC (Roche) for the 18S rDNA probe and anti-digoxigenin rhodamine (Roche) for the Rex1, Rex3 and Rex6 probes. Subsequently, the slides were dehydrated again in an ethanol series (70%, 85% and 100%) for 2 minutes each. After the slides were completely dry, the chromosomes were counterstained with DAPI/Antifading (1.2 mg/ml, Vector Laboratories).

Telomeric (TTAGGG)_n_ repeats were detected using a FITC-labeled PNA probe (DAKO, Telomere PNA FISH Kit/FITC, Cat. No. K5325) according to the manufacturer’s recommendations.

### Microscopy Analyses

Approximately 30 metaphase spreads were analyzed to confirm the diploid chromosome number, karyotype structure and FISH results. Images were captured on an Olympus BX50 microscope (Olympus Corporation, Ishikawa, Japan) using CoolSNAP and the Image Pro Plus 4.1 software (Media Cybernetics, Silver Spring, MD, USA). The chromosomes were classified as metacentric or submetacentric according to their arm ratios [25].

## Results

### Karyotyping and C-banding

**Figure 1:**
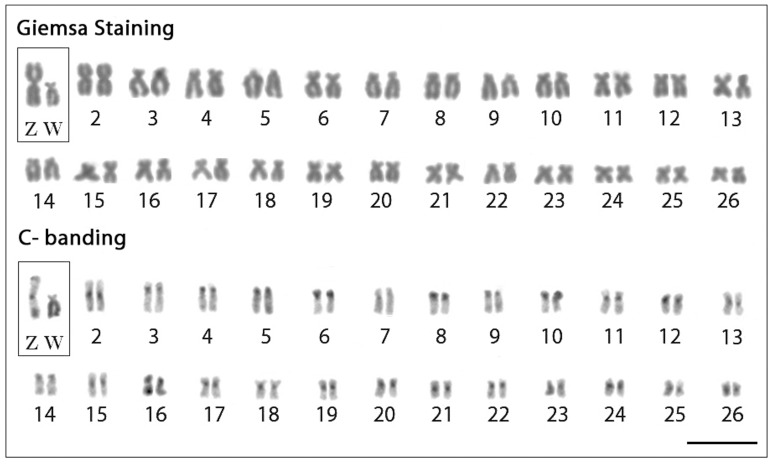
Karyotypes of *Triportheus trifurcatus* female arranged from Giemsa-stained (above) and C-banded chromosomes (below). The ZW sex chromosomes are boxed. Note the conspicuous C-positive heterochromatin accumulated on the W chromosome. Bar = 5 µm.

*T. trifurcatus* showed 2n = 52 chromosomes in both male and female specimens, with the karyotype basically composed of meta/submetacentric chromosomes and a heteromorphic ZZ/ZW sex chromosome system. The Z chromosome is metacentric and the largest of the karyotype, while the W chromosome is submetacentric with a significant reduction in size (Fig. 1). C-positive heterochromatin (C-bands) was observed in the centromeric region of most chromosomes. The Z chromosome also shows additional heterochromatic regions in both telomeres, while the W is almost entirely heterochromatic, except for its small short arm (Fig. 1).

### Chromosomal Mapping of Repetitive Elements

**Figure 2:**
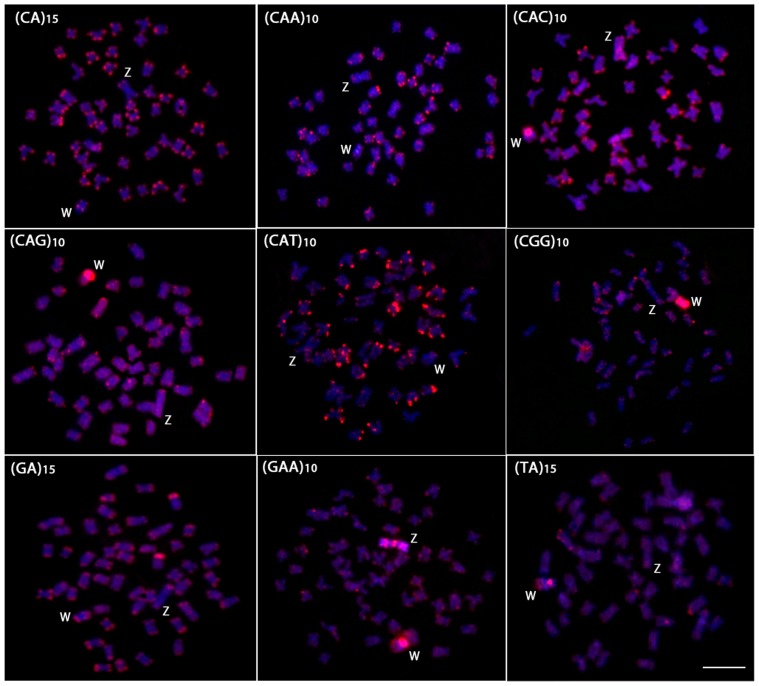
Mitotic metaphase chromosomes of *Triportheus trifurcatus* female hybridized with different labeled microsatellite-containing oligonucleotides. Chromosomes were counterstained with DAPI (blue) and microsatellite probes were directly labeled with Cy3 during synthesis (red signals). Letters mark the Z and W chromosomes. Bar = 5 µm.

**Figure 3:**
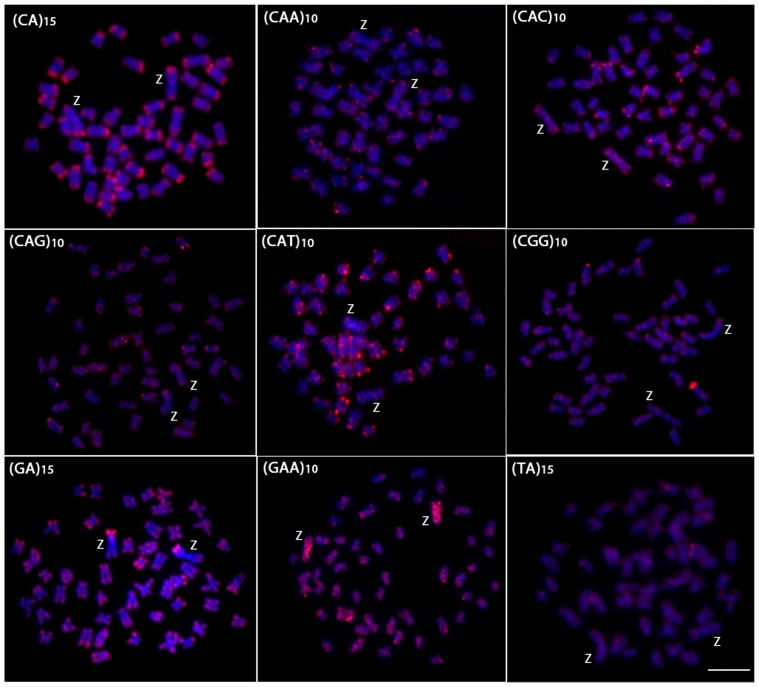
Mitotic metaphase chromosomes of *Triportheus trifurcatus* male hybridized with different labeled microsatellite-containing oligonucleotides. Chromosomes were counterstained with DAPI (blue) and microsatellite probes were directly labeled with Cy3 during synthesis (red signals). Letters mark the Z chromosomes. Bar = 5 µm.

The chromosomal mapping of microsatellite sequences generally showed the same distribution pattern between the autosomes of males and females (Figs. 2 and 3). Most microsatellites were restricted to the telomeric regions, although some presented a more dispersed pattern in both the autosomes and the sex chromosomes. However, the W chromosome differed from the autosomes and the Z chromosome by a large accumulation of four microsatellites. Of these, (CAC)_10_, (CAG)_10_, and (TA)_15_ presented strong signals in the centromeric/pericentromeric region, while the microsatellite (CGG)_10_ accumulated across almost its entire length (Fig. 2). The Z and the W chromosomes showed a strong and dispersed differential accumulation of the microsatellite (GAA)_10_ (Figs. 2 and 3). The microsatellites (CAA)_15_, (CAT)_10_ and (GA)_15_ showed similar hybridization patterns between autosomes and sex chromosomes. Conversely, the microsatellite (CA)_15_ showed interstitial signals in the long arm of the W chromosome, which is different from that observed in the autosomes and Z chromosome, where its accumulation was restricted to telomeric heterochromatin (Figs. 2 and 3).

**Figure 4:**
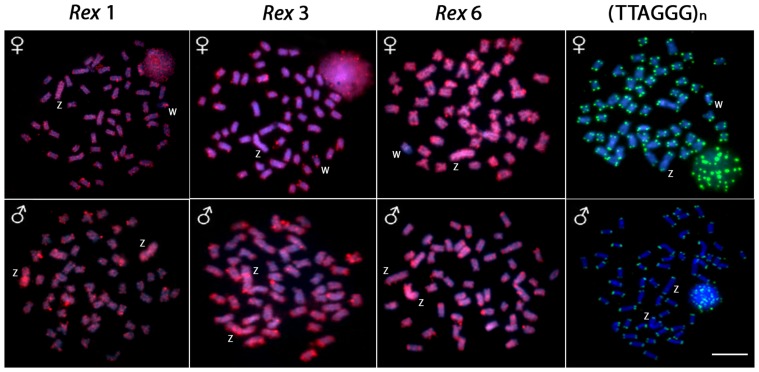
Metaphase plates of *Triportheus trifurcatus* probed with *Rex1*, *Rex3*, *Rex6*, and telomeric (TTAGGG)_n_ sequences. These sequences show the general distribution pattern of these repeats on the chromosomes. Letters mark the Z and W chromosomes. Bar = 5 µm.

**Figure 5:**
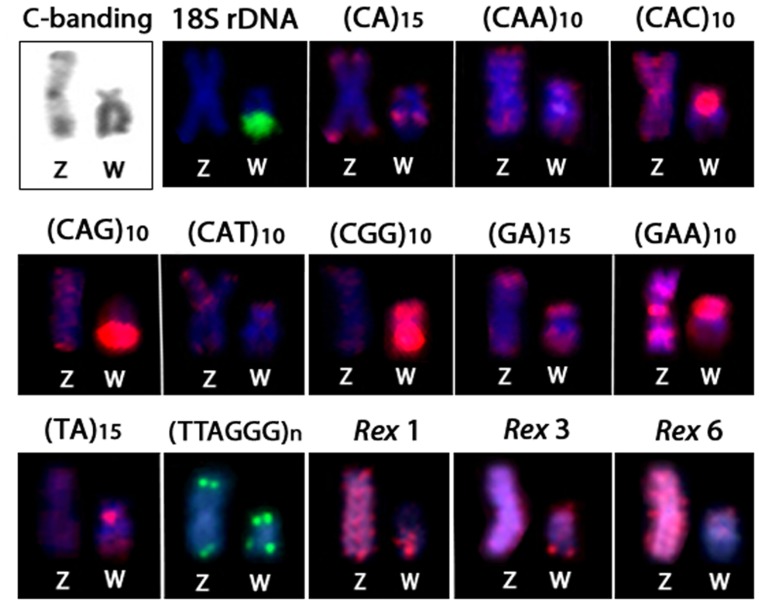
C-banding and the repetitive DNA distribution between the Z and W chromosomes of *Triportheus trifurcatus*. Note the preferential accumulation of heterochromatin and some repeats on the W chromosomes.

The retrotransposons Rex1, Rex3 and Rex6 showed a dispersed distribution on most chromosomes of the complement (including the Z chromosome) and a distribution in small clusters in the telomeric regions of other chromosomes. However, while Rex 3 was widely distributed throughout the whole W chromosome (with more intense accumulation in the telomeric region of the long arms), Rex 1 and 6 preferentially accumulated in the long and short arms of the W chromosome, respectively. The mapping of the telomeric repeats (TTAGGG)n showed signals in both telomeres of all chromosomes (Figs. 4 and 5).

Notably, a conspicuous 18S rDNA cistron occurs on the W chromosome, occupying a large extent of its long arm (Fig. 5). As a result, in all FISH experiments, the 18S rDNA probe was simultaneously applied to correctly identify the W chromosome (data not shown).

## Discussion

One of the steps for sex chromosomes evolution is the accumulation of repetitive DNAs that is accompanied by a heterochromatinization process. The accumulation of repetitive elements on the sex chromosomes is favored by the absence or low frequency of recombination, which is caused by the structural and/or DNA changes that occur during the sex chromosome’s differentiation process [26]. Despite this general rule, consensus indicates that not all events involved in the differentiation of the Y chromosome are necessarily valid for W chromosome differentiation [12], [13]. In fact, sex chromosome evolutionary mechanisms occurred independently among lineages, including plants, insects, fishes, birds and mammals [27]. Among fishes, this scenario is evident because the W chromosome presents different morphologies and sizes and, in some cases, is larger than the Z chromosome [28]–[31].

The heterochromatinization of the W chromosome that occurs in *T. trifurcatus* is also observed in other species of this genus, but with distinct distribution patterns. In contrast, the Z chromosomes of all species show the conserved feature of heterochromatin that is present only in both telomeric regions [7], [11]. Moreover, the occurrence of a conspicuous 18S rDNA cistron on the W chromosome may correspond to a shared condition in *Triportheus* because all of the species analyzed to date showed this characteristic [8], [32], with the exception of *T. venezuelensis* [9]. Certainly, the increase in heterochromatin has driven the differentiation of the W chromosome, as also occurs in other ZW systems in fishes such as *Thoracocharax*, *Parodon*, *Leporinus* and *Apareiodon* [12]. Additionally, the absence of interstitial (TTAGGG)n signals demonstrates that the origin of the sex system in this species did not involve rearrangements such as fusions and fissions, which is different from the scenario found in the formation of multiple sex chromosomes reviewed in [33] (Fig. 4).

In addition to studies related to genes linked to the sex chromosomes, other research has been conducted on the mapping of repetitive DNA sequences. These approaches verifed through differential accumulation that these sequences have a prominent role in the evolution of sex-specific chromosomes [23], [33]. Modern cytogenetic techniques that are more sensitive than C-banding have enabled the characterization of sex chromosomes across a wider range of species [13]. The FISH technique clearly demonstrated that some microsatellites accumulated preferentially in the heterochromatic region of the W chromosome, highlighting their probable role in the differentiation of these chromosomes (Figs. 2 and 5). This same feature was observed in other *Triportheus* species, such as *T. auritus*, where a large accumulation of several microsatellites was found on the W chromosome [34]. In *T. auritus*, only the (C)_30_ microsatellite accumulated equally in both the W chromosome and the autosomes, while eight other microsatellites showed a large accumulation in the W chromosome, particularly in the heterochromatic regions, but not in other chromosomes. Concerning the Z chromosome, the distribution of the microsatellite repeats was similar to that found in the autosomes, thus reinforcing the hypothesis that the differential microsatellite accumulation between the Z and W was an essential step for the differentiation of the sex pair in *Triportheus* species.

In other fish species, microsatellites also preferentially accumulated in the heteromorphic sex chromosomes. In fact, several microsatellites showed preferential accumulation in the heterochromatic region of the W chromosome in *Leporinus* species [35]. Similarly, the same pattern observed for the microsatellite (GAA)_10_ in *T. trifurcatus* was observed in the wolf fish *H. malabaricus,* with a similar accumulation in both the X and Y chromosomes [36]. Thus, it is clear that the accumulation of microsatellites plays an important role in sex chromosome differentiation. However, microsatellites can present identical or different distribution patterns on the sex chromosomes of different species. For example, while microsatellites accumulate preferentially in the sex-specific chromosome in some species, in others, they have a widespread distribution among all karyotypes [22], [36], [37]. Indeed, microsatellites are dynamic components of the genome and are formed by distinct molecular processes such as DNA replication slippage or the insertion or deletion of one or more repeats that can also drive their genomic accumulation and distribution [38], [39].

Transposable elements have the ability to copy or transpose themselves into non-homologous regions of a genome. These sequences can also show different evolutionary dynamics that result in small to large genomic fractions [17]. Among many retrotransposons, the *Rex* family has been widely analyzed in different fish species because its family members are abundant, and their distribution varies from a scattered pattern to preferential accumulation in certain regions of the chromosomes [reviewed in 19]. Our present data show that the distinct *Rex* elements accumulated in different ways in the W and in the other chromosomes of *T. trifurcatus* (Fig. 4). Interestingly, instead of presenting the typical pattern of microsatellites, i.e., accumulation in the W chromosome, *Rex*6 sequences were restricted to a few clusters in the W chromosome. As for the *Rex*1 and *Rex*3 elements, no differential accumulation between the W and the other chromosomes was observed, suggesting that, at least for this TE family, their evolutionary dynamics may be more diverse than previously assumed.

The analysis of the genomic composition of several microsatellites and retrotransposons in the fish *T. trifurcatus* helps us better understand the correlation of repetitive DNAs with the differentiation of the ZW sex pair. A strict correlation has clearly been shown between the differentiation of the sex-specific W chromosome and the accumulation of several DNA repeats, resulting in the heterochromatinization of this chromosome.

## References

[1] Oliveira C, Foresti F, Hilsdorf AWS (2009) Genetics of neotropical fish: from chromosomes to populations. Fish Physiol Biochem 35: 81–100. 18683061 10.1007/s10695-008-9250-1

[2] Devlin RH, Nagahama Y (2002) Sex determination and sex differentiation in fish: an overview of genetic, physiological, and environmental influences. Aquaculture. 208: 191–364.

[3] Malabarba MCSL (2004) Revision of the Neotropical genus *Triportheus* Cope, 1872 (Characiformes: Characidae). Neotrop Ichthyol 2: 167–204.

[4] Falcão JN (1988) Caracterização cariotípica em peixes do gênero *Triportheus* (Teleostei, Characiformes, Characidae). PhD Thesis, Universidade de São Paulo, Ribeirão Preto, SP, Brazil.

[5] Bertollo LAC, Cavallaro ZI (1992) A highly differentiated ZZ/ZW sex chromosome system in a Characidae fish, *Triportheus guentheri* . Cytogenet Cell Genet 60: 60–63. 1582261 10.1159/000133296

[6] Sánchez S, Jorge LC (1999) A new report of the ZZ/ZW sex chromosome system in the genus *Triportheus* (Pisces, Triportheinae). Cytologia 64: 395–400.

[7] Artoni RF, Falcão JN, Moreira-Filho O, Bertollo LAC (2001) An uncommon condition for a sex chromosome system in Characidae fish. Distribution and differentiation of the ZZ/ZW system in *Triportheus*. Chromosome Res. 9: 449–456. 10.1023/a:1011620226348 11592479

[8] Artoni RF, Bertollo LAC (2002) Evolutionary aspects of the ZZ/ZW sex chromosome system in the Characidae fish, genus *Triportheus*. A monophyletic state and NOR location on the W chromosome. Heredity 89: 15–19. 12080365 10.1038/sj.hdy.6800081

[9] Nirchio M, Oliveira C, Ferreira IA, Granado A, Ron E (2007) Extensive polymorphism and chromosomal characteristics of ribosomal DNA in the characid fish *Triportheus venezuelensis* (Characiformes, Characidae). Genet Mol Biol 30: 25–30.

[10] Diniz D, Laudicina A, Cioffi MB, Bertollo LAC (2008) Microdissection and whole chromosome painting. Improving sex chromosome analysis in *Triportheus* (Teleostei, Characiformes). Cytogenet Genome Res 122: 163–168. 19096212 10.1159/000163094

[11] Diniz D, Moreira-Filho O, Bertollo LAC (2008) Molecular cytogenetics and characterization of a ZZ/ZW sex chromosome system in *Triportheus nematurus* (Characiformes, Characidae). Genetica 133: 85–91. 17705059 10.1007/s10709-007-9187-9

[12] Cioffi MB, Camacho JPM, Bertollo LAC (2011) Repetitive DNAs and the differentiation of sex chromosomes in Neotropical fishes. Cytogenet Genome Res 132: 188–194. 21042005 10.1159/000321571

[13] Ezaz T, Sarre S, O’Meally D, Graves JAM, Georges A (2009) Sex Chromosome Evolution in Lizards: Independent Origins and Rapid Transitions. Cytogenet Genome Res 127: 249–260. 20332599 10.1159/000300507

[14] Ohno S (1967) Sex chromosomes and sex-linked genes. Springer, Berlin Heidelberg New York.

[15] Charlesworth B, Snlegowski P, Stephan W (1994) The evolutionary dynamics of repetitive DNA in eukaryotes. Nature 371: 215–220. 8078581 10.1038/371215a0

[16] Lippman Z, Gendrel AV, Black M, Vaughn MW, Dedhia N, et al (2004) Role of transposable elements in heterochromatin and epigenetic control. Nature 430: 471–476. 15269773 10.1038/nature02651

[17] Schueler MG, Higgins AW, Rudd MK, Gustashaw K, Willard H (2001) Genomic and genetic definition of a functional human centromere. Science 294: 109–115. 11588252 10.1126/science.1065042

[18] Biémont C, Vieira C (2006) Junk DNA as an evolutionary force. Nature 443: 521–524. 17024082 10.1038/443521a

[19] Cioffi MB, Bertollo LAC (2012) Chromosomal Distribution and Evolution of Repetitive DNAs in Fish. In: Garrido Ramos, editor. Repetitive DNAs Genome Dynamics. Basel: Karger, 197–221. 10.1159/000337950 22759820

[20] Bertollo LAC, Takahashi CS, Moreira-Filho O (1978) Cytotaxonomic considerations on *Hoplias lacerdae* (Pisces Erythrinidae). Braz J Genet 2: 103–120.

[21] Sumner AT (1972) A simple technique for demonstrating centromeric heterochromatin. Exp Cell Res 75: 304–306. 4117921 10.1016/0014-4827(72)90558-7

[22] Kubat Z, Hobza R, Vyskot B, Kejnovsky E (2008) Microsatellite accumulation in the Y chromosome of *Silene Latifolia.* . Genome 51: 350–356. 18438438 10.1139/G08-024

[23] Volff JN, Korting C, Sweeney K, Schartl M (1999) The non-LTR retrotransposon *Rex3* from the fish *Xiphophorus* is widespread among teleosts. Mol Biol Evol 16: 1427–1438. 10555274 10.1093/oxfordjournals.molbev.a026055

[24] Cioffi MB, Martins C, Centofante L, Jacobina U, Bertollo LAC (2009) Chromosomal variability among allopatric populations of Erythrinidae fish *Hoplias malabaricus*: mapping of three classes of repetitive DNAs. Cytogenet Genome Res 125: 132–141. 19729917 10.1159/000227838

[25] Levan A, Fredga K, Sandberg AA (1964) Nomenclature for centromeric position on chromosomes. Hereditas 52: 201–220.

[26] Charlesworth D, Charlesworth B, Marais G (2005) Steps in the evolution of heteromorphic sex chromosomes. Heredity 95: 118–128. 15931241 10.1038/sj.hdy.6800697

[27] Vallender EJ, Lahn BT (2004) How mammalian sex chromosomes acquired their peculiar gene content. BioEssays 26: 159–169. 14745834 10.1002/bies.10393

[28] Feldberg E, Bertollo LAC, Almeida-Toleto LF, Foresti F, Moreira-Filho O, et al (1987) Biological aspects of Amazonian fishes. IX. Cytogenetic studies in two species of the genus *Semaprochilodus* (Pisces,Prochilodontidae). Genome 29: 1–4.

[29] Venere PC, Ferreira IA, Martins C, Galetti PM Jr (2004) A novel ZZ/ZW sex chromosome system for the genus *Leporinus* (Pisces, Anostomidae, Characiformes). Genetica 121: 75–80. 15098739 10.1023/b:gene.0000019936.40970.7a

[30] Silva DS, Milhomem SSR, Pieczarka JC, NagamachiI CY (2009) Cytogenetic studies in *Eigenmannia virescens* (Sternopygidae, Gymnotiformes) and new inferences on the origin of sex chromosomes in the *Eigenmannia* genus. BMC Genet. 10: 74. 10.1186/1471-2156-10-74 PMC2789750 19930594

[31] Bellafronte E, Schemberger MO, Artonni RF, Moreira-Filho O, Vicari MR (2012) Sex chromosome system ZZ/ZW in *Apareiodon hasemani* Eigenmann, 1916 (Characiformes, Parodontidae) and a derived chromosomal region. Genet Mol Biol 35: 770–776. 23271937 10.1590/S1415-47572012005000077 PMC3526084

[32] Marquioni V, Bertollo LAC, Diniz D, Cioffi MB (2013) Comparative chromosomal mapping in *Triportheus* fish species. Analysis of synteny between ribosomal genes. Micron 45: 129–135. 23273577 10.1016/j.micron.2012.11.008

[33] Cioffi MB, Moreira-Filho O, Almeida-Toledo LF, Bertollo LAC (2012) The contrasting role of heterochromatin in the differentiation of sex chromosomes: an overview from Neotropical fishes. J Fish Biol 80: 2125–2139. 22551173 10.1111/j.1095-8649.2012.03272.x

[34] Cioffi MB, Kejnovský E, Marquioni V, Poltronieri J, Molina WF, et al (2012) The key role of repeated DNAs in sex chromosome evolution in two fish species with ZW sex chromosome system. Mol Cytogenet 5: 28. 22658074 10.1186/1755-8166-5-28 PMC3462698

[35] Poltronieri J, Marquioni V, Bertollo LAC, Kejnovsky E, Molina WF, et al (2013) Comparative chromosomal mapping of microsatellites in *Leporinus* species (Characiformes, Anostomidae): Unequal accumulation on the W chromosomes. Cytogenet Genome Res 142 (1): 40–5. 10.1159/000355908 24217024

[36] Cioffi MB, Kejnovsky E, Bertollo LAC (2011) The Chromosomal Distribution of Microsatellite Repeats in the Genome of the Wolf Fish *Hoplias malabaricus*, Focusing on the Sex Chromosomes. Cytogenet Genome Res 132: 289–296. 21099206 10.1159/000322058

[37] Pokorná M, Kratochvíl L, Kejnovský E (2011) Microsatellite distribution on sex chromosomes at different stages of heteromorphism and heterochromatinization in two lizard species (Squamata: Eublepharidae: *Coleonyx elegans* and Lacertidae: *Eremias velox*). BMC Genet 12: 90. 22013909 10.1186/1471-2156-12-90 PMC3215666

[38] Levinson G, Gutman GA (1987) Slipped-strand mispairing: a major mechanism for DNA sequence evolution. Mol Biol Evol 4 (3): 203–221. 10.1093/oxfordjournals.molbev.a040442 3328815

[39] Ellegren H (2000) Microsatellite mutations in the germline: implications for evolutionary inference. Trends Genet 16: 551–558. 11102705 10.1016/s0168-9525(00)02139-9

