# Correction: Chromosomal Mapping of Repetitive DNAs in *Triportheus trifurcatus* (Characidae, Characiformes): Insights into the Differentiation of the Z and W Chromosomes

**DOI:** 10.1371/journal.pone.0100494

**Published:** 2014-06-12

**Authors:** The PLOS ONE Staff

There is an error in the author affiliations for Roberto Ferreira Artoni and Thomas Liehr. The correct affiliations for these authors are:

Roberto Ferreira Artoni - Departamento de Biologia Estrutural, Molecular e Genética, Universidade Estadual de Ponta Grossa, Ponta Grossa, PR, Brazil

Thomas Liehr – Jena University Hospital, Friedrich Schiller University, Institute of Human Genetics, Jena, Thüringen, Germany.

## References

[1] Yano CF, Poltronieri J, Bertollo LAC, Artoni RF, Liehr T, et al (2014) Chromosomal Mapping of Repetitive DNAs in *Triportheus trifurcatus* (Characidae, Characiformes): Insights into the Differentiation of the Z and W Chromosomes. PLoS ONE 9(3): e90946 doi:10.1371/journal.pone.0090946 24632562 10.1371/journal.pone.0090946 PMC3954618

